# Navigating Community-Based Psychiatry Care of Pediatric Patients in Low-Resource Settings: A Case Report

**DOI:** 10.7759/cureus.59506

**Published:** 2024-05-02

**Authors:** Natalie Regian, Ajay Mittal, Michael Chammany, Hitesh P Rai, Michael Rommen

**Affiliations:** 1 Medical School, Edward Via College of Osteopathic Medicine, Monroe, USA; 2 Nephrology, University of Florida College of Medicine, Gainesville, USA; 3 Research, Edward Via College of Osteopathic Medicine, Monroe, USA; 4 Clinical Medicine/Emergency Medicine, Edward Via College of Osteopathic Medicine, Monroe, USA; 5 Emergency Medicine, St. Francis Medical Center, Monroe, USA

**Keywords:** autism spectrum disorder and emotion, mental health services, pediatric emergency department (ped), access to healthcare, child and adolescent psychiatry

## Abstract

This case report examines the experience of a nine-year-old male with autism spectrum disorder (ASD) who was admitted to his rural community emergency department (ED) for the treatment of aggressive behaviors, agitation, and violence. Despite a high prevalence of such behaviors within the autistic pediatric population, multiple inpatient facilities that offer pediatric psychiatric services refused to transfer his care. Many other commonly used resources and treatment modalities were also not available, resulting in a nine-day ED boarding experience with minimal symptomatic improvement. Pharmacotherapy was utilized, but nationally recommended guidelines were not appropriately followed. Although this case is one of many pediatric patients who received inadequate psychiatric care from their local ED, it is significant in identifying specific areas of improvement within Northeast Louisiana psychiatric healthcare. This case report of a nine-year-old male with autism underlines the hardships faced by patients and their families due to the gaps in our current healthcare infrastructure and emphasizes the importance of protocols and resources for patient populations with higher-than-average wellness needs.

## Introduction

Autism spectrum disorder (ASD)

ASD is a neurodevelopmental disorder characterized by deficits in social communication and the presence of restricted interests and repetitive behaviors [1]. Since the publication of the Diagnostic and Statistical Manual of Mental Disorders-5th edition (DSM-5) in 2013, severity-level descriptors have been added to the diagnosis of ASD to better categorize the level of support needed by an individual with autism. Therefore, the diagnosis of ASD is followed by one of the subsequent specifiers: requiring very substantial support, requiring substantial support, and requiring support [2]. Approximately 1/100 children are diagnosed with ASD around the world as early as 18-24 months of age, typically presenting with initial symptoms of delayed language, cognitive, or movement skills [1,3]. Common characteristics related to ASD include lack of displayed emotion, repetitive meaningless gestures, lack of interaction with others, obsessive interests, emotional lability related to minor changes, unusual eating and sleeping habits, and impulsive/inattentive behavior [4]. 

The most widely accepted and practiced treatment for maladaptive behaviors in those with autism is applied behavioral analysis therapy (ABA) [5]. ABA utilizes the psychological principles of learning theory to bring about change in unwanted behaviors, subsequently improving social interactions [6]. Second to therapeutic intervention, pharmacologic options for ASD-associated irritability have become readily available. Second-generation antipsychotics (SGAs) are the first-line medication options for aggression in ASD, with risperidone and aripiprazole specifically approved by the FDA to treat irritability in this population [7]. Following several supportive studies, anti-epileptic drugs (AEDs) are more frequently prescribed in an off-label fashion to target irritability and disruptive mood symptoms in children with ASD [7]. If aggressive behavior persists past these options, a patient is considered refractory. In one study, 39.5% of ASD patients met the criteria for drug-refractory behaviors after the trials of three or more psychotropic drugs known to target irritability [8,9]. No guidelines exist for the treatment of refractory ASD-associated aggression. Novel approaches to treatment are beginning to surface, including the investigation of glutamatergic agents and gamma-aminobutyric acid modulators, but evidence is not yet available to support their use [5].

Access to psychiatric care

Using nationwide data that spanned from 2007 to 2016, Lo et al. (2020) assessed the emergency department (ED) visits made by children aged 5-17 years with a mental health disorder [10]. Most notably, they concluded that most of these visits occurred at nonchildren’s facilities in metropolitan and rural settings, both proving to be less prepared for the high level of care demanded by this population than larger urban regions. Healthcare centers who see fewer pediatric patients (less than 4000 annually) also rank among the less provisioned [10,11]. Considering these circumstances, the treatment routes following a pediatric psychiatry patient’s visit to the emergency room are limited. When inpatient hospital admission is warranted due to the severity of aggression, self-harm behaviors, or other qualifying symptomatology, many patients experience extensive periods of ED boarding, typically exceeding 24 hours [12]. Reasons for delay in admission to inpatient psychiatry include unawareness of illness, explanatory models of illness, stigma, and financial constraints [13]. ED boarding is defined as the temporary holding of an admitted patient specifically in the ED, as opposed to a more stable location in the hospital, while awaiting an inpatient room and bed. In regard to pediatric-specific policies or procedures, the National Pediatric Readiness Project concluded that only 73.1% of EDs had mental health care plans, 66.5% had guidelines for behavioral health transfers, 67.2% had social service plans, 62.5% of EDs had resources for family-centered care planning, and 71.7% held interfacility agreements and guidelines for the transfer of pediatric patients [14]. 

We report a case of a nine-year-old male who presented to the ED due to aggressive behavior secondary to ASD and attention-deficit hyperactivity disorder (ADHD) in Northeast Louisiana. This case highlights the limitations patients and emergency rooms endure when managing psychiatric emergencies in the United States.

## Case presentation

A nine-year-old overweight, African American male presented to a community ED via emergency medical services due to aggressive behavior. The patient had a past medical history of ASD, ADHD-combined type, macrocephaly, learning and intellectual disability, and developmental sleep disorder. The patient had a history of being hospitalized due to behavioral issues. During this visit to the ED, the patient was accompanied by his mother, and his family reported that the patient had been quickly oscillating between calmness and physically attacking those around him. He also displayed self-injurious behaviors, scratching, and hurting himself frequently without successful redirection for several hours. He was nonverbal, only communicating through sounds and gestures without coherent speech. He denied abdominal pain and headaches, and his mother denied any signs of physical distress, aches, or pains. The patient’s mother stated that the patient did not use tobacco, alcohol, or any other illicit drugs. The patient’s mother stated that she and the patient’s ABA therapist noticed that he would often blank out and stare into space; she was unable to quantify the duration and frequency of these episodes. The patient was under the care of a psychiatric nurse practitioner and pediatric neurologist, and he was awaiting a video electroencephalogram (VEEG) due to concern of possible seizures.

On physical exam, the patient appeared to be stable, with the only concerning physical finding being a red excoriation mark on his right cheek with no bleeding and scattered, small scabs on his left arm. The patient was noncommunicative and refused to make eye contact with the provider, but he was cooperative. The provider ensured that the patient was constantly monitored, and a comprehensive metabolic panel (CMP), urine analysis (UA), complete blood count (CBC), drug panel, and COVID-19 test were ordered; no significant findings were seen based on the labs ordered. Due to intermittent aggressive behavior (hitting himself repeatedly, posturing toward others, failing to obey commands, damaging property, etc.), the patient was given lorazepam 1 mg, and two hours later, he was given haloperidol 2.5 mg for continuing aggressive behavior. A Physician’s Emergency Certificate (PEC) was signed to ensure that the patient would receive the care that he needed, and he was cleared to be transferred to a psychiatric hospital. The referral was denied two hours later due to the psychiatric hospital stating that it would not be able to take care of the patient due to his aggressive behavior.

In the following days, the patient’s social worker first attempted to contact the patient’s outpatient psychiatric nurse practitioner (NP). The NP informed the social worker that the patient had seen two psychiatrists and did not respond to psychiatric medications. The social worker and the NP attempted to contact several other psychiatric hospitals in an attempt to continue care out of the ED, but the patient was repeatedly denied, citing reasons such as the inability to manage the patient’s violent behavior and lack of staffing. Meanwhile, he was given risperidone routinely and clonidine and haloperidol as needed to manage his aggressive outbursts in the ED. Throughout his second day in the emergency room, the patient was given risperidone 0.5 milligrams (mg), two doses of clonidine 0.2 mg, and haloperidol lactate 2 mg to manage his aggression; for the following days, the patient was given risperidone 1 mg once per day for seven days and additional doses of 1 mg were given as needed. Two days after presenting to the ED, the patient’s Medicaid agreed to a single-case agreement for out-of-state referral if all in-state options were exhausted. Four days after presenting to the ED, the social worker proposed placing the patient in a group home due to lack of facilities willing to take care of this patient. The patient’s mother refused, stating “I just can’t give my baby up. If you all cannot find a medication to help him, we will figure it out.” The provider refused to discharge the patient due to his self-injurious behaviors, violence toward others, and necessity of higher level of care, and the social worker contacted child protective services (CPS) to report the situation.

**Figure 1:**
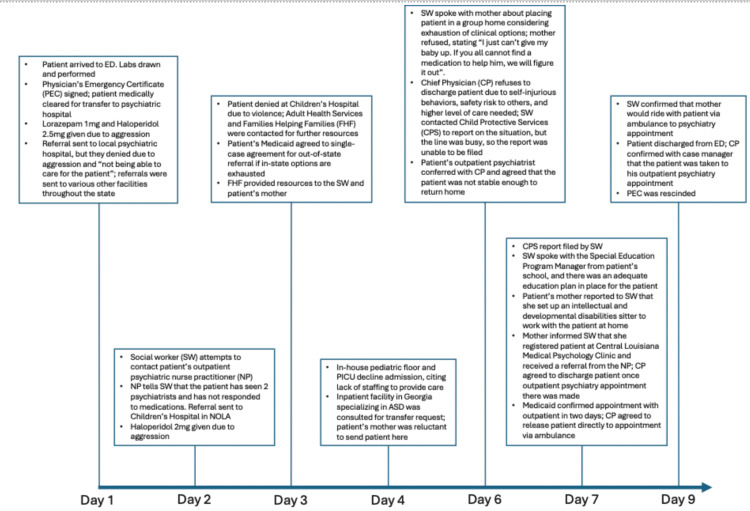
Overall care timeline and chart dialogue

Six days after presenting to the ED, the patient was finally able to establish outpatient psychiatric care per Medicaid confirmation. The patient’s mother also confirmed that she had set up an intellectual and developmental disabilities (IDD) sitter to work with the patient at home, while the patient’s school reported that there was an adequate education plan in place for him following discharge. With these plans in place, the ED physician agreed to discharge the patient from the ED via ambulance directly to the outpatient appointment. On day nine of his stay in the hospital, the patient was discharged from the ED. The overall care timeline and chart dialogue is illustrated in Figure 1. The patient’s PEC was rescinded, and he was transported with his mother via ambulance to the psychiatry appointment.

## Discussion

**Figure 2:**
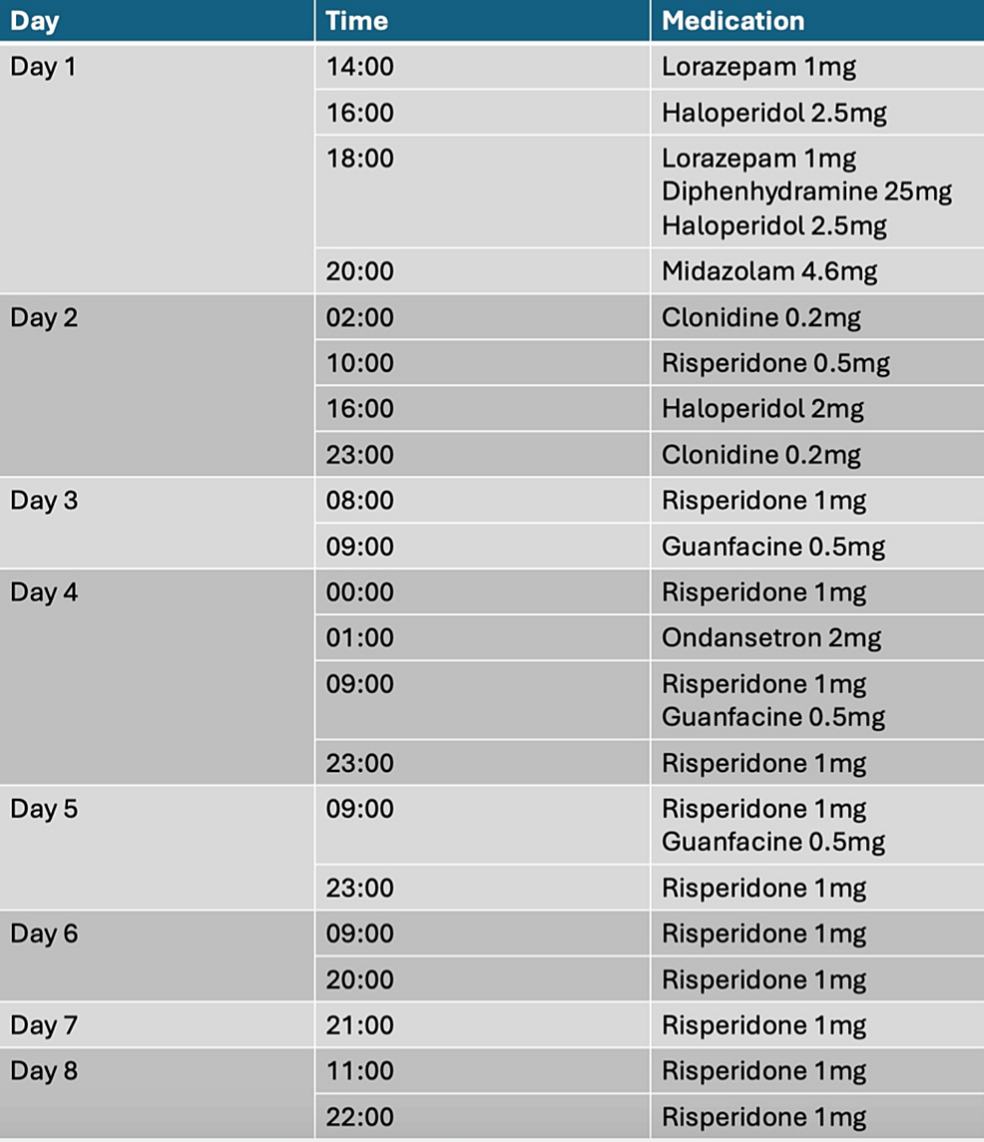
Medications administered during the course of hospitalization

The patient presented in this case report boarded in the local ED for eight nights/nine days, significantly exceeding the Joint Commission’s recommendation of only four hours [15]. During this time, he was administered seven different medications to address his aggressive behaviors and violent outbursts, as detailed in Figure 2. However, two of these medications, lorazepam and midazolam, have not been approved for on-label use of agitation or aggression in the pediatric population. Despite some reported success of benzodiazepine use in children to resolve acute-onset aggressive behaviors, these drugs are used cautiously in children with autism because of their potential for disinhibition [16]. Antipsychotics are the recommended first-line medications for managing difficult behaviors in this patient population, yet haloperidol was the only antipsychotic used on the patient’s first day of admission and not until after two failed doses of lorazepam. Positively, and as condoned by the FDA, risperidone was appropriately and frequently administered to the patient to combat his undesired behaviors. There is no documentation of behavioral improvement until day four of his ED boarding. Between days one and four, the patient received five doses of risperidone. This was the most consistent and most frequently administered anti-aggression pharmacotherapy provided to him, indicating a potential correlation between this drug and his improved volatility. AEDs were never used throughout his course in the ED despite support for this drug-indication combination in the literature. The concurrent use of multiple antipsychotics was not attempted although this is noted to be a frequent practice in drug-resistant and refractory agitation. The patient was also not continued on his home medications throughout his onboarding despite one of these (aripiprazole) being FDA-approved to treat aggression in his population subset [5]. The patient’s home medications included aripiprazole (Abilify) 1 mg/mL liquid, 10 mL by mouth in the morning and 10 mL by mouth at bedtime, Azstarys 39.2 mg-7.8 mg capsule, one capsule by mouth every morning, and clonidine 0.2 mg tablet, one tablet by mouth at bedtime.

Considering that within this rural, nonmetro community of 46,820 people only 25.3% are reported to be under 18 years of age, it is not surprising that the local hospital does not employ many pediatric-specific healthcare personnel [17]. As noted previously by McBride, a low volume of pediatric caseloads typically results in lower availability of pediatric resources and specialists [12]. Therefore, it is unsurprising that the case manager and social worker involved in the patient’s case are not pediatric care specialists. The emergency medicine physicians who attended to him daily in the ED are not pediatric emergency medicine specialists. The presiding hospital does not have a psychiatrist of any subspecialty employed full-time, subsequently negating the possibility of an in-house consult. Nonetheless, no part-time, locums, or telemedicine psychiatrist was ever consulted to evaluate the patient. The patient was provided with a line-of-site aid for continual safety monitoring but had minimal interaction with this rotating employee. He also did not receive any educational instruction throughout the seven weekdays in which he was boarded, and his visitation time with family members was limited, both by ED policy and familial availability. He did not receive any mental health counseling, recreational activity time, socialization with peers, or individualized therapy. Web-based assessments/evaluations and outpatient visits, family-centered interventions, and home-based care were not offered to the patient or his caregivers. Requests for transfer of care were sent appropriately with inappropriate denials of the patient at multiple facilities. Most attributed their decision to his history/presentation of aggression and violence despite the common presentation of aggression and maladaptive behaviors in children with ASD. CPS was rightly notified of the patient’s precarious situation, limited resources, and discordant guardian. Social work was able to provide the patient’s primary caregiver with an outpatient resource that would assist her burden at home by supplying the patient with an IDD sitter/behavioral assistant. The shortage of mental health facilities catering to specialized care for pediatric patients presented in this case highlights the challenges faced in providing adequate mental health services for young patients.

## Conclusions

The patient presented in this case report received inadequate care to address his and his family’s concerns regarding the agitation, aggression, and injurious behaviors provoked and exacerbated by autism. As a pediatric psychiatric patient with an accompanying neurodevelopmental disorder, his local ED and managing hospital did not have the resources necessary for providing him with substantial psychiatric care. There is a need for further research to better identify barriers to access to care for psychiatric patients of all ages to support evidence-based practices to be incorporated into routine care for this patient population when in distress. There is a considerable burden placed on families due to prolonged hospitalizations both medically and psychologically; much of which could be reduced with specialists who are equipped to treat child psychiatry complaints appropriately. In light of the largely negative and unsupportive experience with a rural ED, this patient’s case emphasizes the outstanding need for accessible pediatric psychiatric treatment resources and highlights a region and population of the country desperate for attention and improvement.
